# JNS Review Recognition 2018

**DOI:** 10.1017/jns.2018.24

**Published:** 2018-11-23

The Editorial Board of the *Journal of Nutritional Science* would like to thank the following for their contribution as peer reviewers in 2018.

Folasade Adebayo
Karen Assmann
Nuri Aykan
Claire Berryman
Maria Bouga
Kathryn Bradbury
Louise Brough
Aline Lopes Bueno
Ruud Buijs
Elisabet Børsheim
Maria Cavaletto
Ling-Wei Chen
Marike Cockeran
Vanessa de Mello Laaksonen
Alison Eldridge
Emily Farina
Emma Feeney
Michael Flock
Kurt Gedrich
Eileen Gibney
Ryan Grant
William Grant
Ingibjörg Gunnarsdóttir
Ella Haddad
Neil Harris
Christina Hartmann
Alyson Hill
Adela Hruby
Jasminka Ilich-Ernst
Geert Janssens
Hana Kahleova
Rajeev Kapila
Toshiyuki Kohri
Wojciech Krężel
Venu Lagishetty
Ashley Lederer
Alberto Leguina
Kenneth Lo
Bohdan Luhovyy
Kelsey Mangano
Koutatsu Maruyama
Thomas Mason
Anine Medin
Charlotte Mortensen
Ana Carolina Mosca
Kentaro Murakami
Sohail Mushtaq
Ran Nir-Paz
Camila Oliveira
Sue Penckofer
Stefany Primeaux
Gail Rees
Caroline Richard
Christian Ritz
Luis Romero
Mayra Saleh
Jay Schulkin
Barbara Korousic Seljak
Shigenobu Shibata
Barbara Shukitt-Hale
Kylie Smith
Christine Sommer
Matúš Soták
Xiong Su
Priya Sumithran
Izabela Szczerbal
Norman Temple
Sam Thiagalingam
Tammy Tong
Paul Trayhurn
Hans Van Trijp
Vaclav Vetvicka
Carol Wagner
Ben Wheeler
Philip Whitfield
Corné van Dooren
Linde van Lee

